# 

**Published:** 1841-05

**Authors:** 


### CONTENTS

OF NO. V.

---

### ORIGINAL COMMUNICATIONS.

### ESSAYS AND CASES.

Art. I.—A Memoir on Obliteration of the Bronchiæ. By A. C. Reynaud, M. D. formerly resident in the Hospital; late Chief of Clinical Medicine of the Paris Faculty; Physician at Puy, &c. Translated for the Western Journal of Medicine and Surgery: By John A. Warder, M. D., of Cincinnati. - - - - - - - - - 321

Art. II.—Utility of Tonico-Astringent Injections into a Suppurative Abscess. A Case. By Norvin Green, M. D. of Bedford, Ky. - - - - - - - - 343

Art. III.—History of a Case of Autumnal Fever; accompanied by the vomiting of a fluid of a deep blue color. By George K. Holloway, M. D., of Panola, Mississippi. 347

Art. IV.—A Case of Hepatic Abscess, in which Biliary Calculi were discharged at the side, after an operation resulting favorably. By William Dawson Dorris, M. D., of Nashville, Tennessee. - - - - - - 352

Art. V.—Case of Pretended Mayhem, which was tried before the Mayor's Court in Cincinnati, March, 1840. By S. D. Gross, M. D. - - - - - - - - 355

REVIEWS.

ART. I.—Du Varicocele, et en particulier de la Cure Radicale de Cette Affection; par H. LANDOUZY, interne a l'Hotel-Dieu, membre de la Societe Anatomique et de la Societe Medicale D'Observation, &c. - - - - - 357
ART. II.—Manuel de Medicine Operatoire, fonde sur L'Anatomie Normale et L'Anatomie Pathologique. Par J. F. MALGAIGNE, M. D. - - - - - - - - - 371
ART. III.—J. F. HENKEL's Anleitung zum Chirurgischen Verbande. Umgearbeitet und mit vielen Zusatzen versehen von Dr. J. C. Stark. Von neum bearbeitet und mit Zusatzen vermehrt von J. F. Dieffenbach, M. D. - - - 379
Bibliographical Notice. - - - - - - 381

SELECTIONS FROM AMERICAN AND FOREIGN JOURNALS.

On a New Species of Torticollis. By M. BOUVIER. - - - 383
On a New Method of Operating for the Radical Cure of Hernia. By M. VELPEAU. - - - - - - - - - 384
Rhinoplastic and Cheiloplastic Operations. - - - - - 385
Anatomical Description of an Hermaphrodite. By Prof. MAYOR, of Bon. - - - - - - - - - - - 386
New Method for the Radical Cure of Varix, and especially of Varicocele. By M. RICORD. - - - - - - - 393
Congenital Dislocation of the Humerus reduced after sixteen years. By M. GAILLARD, Surgeon to the Hotel Dieu, of Poictiers. 395

ORIGINAL INTELLIGENCE.

A Card. - - - - - - - - - - 397
Medical Convention of Ohio. - - - - - - - - 397
Resignations, Appointments and Deaths in Medical Schools. 397
Professor Caldwell. - - - - - - - - - 398
Death of President Harrison, - - - - - - - 398
Medical Hoax. - - - - - - - - - - 399
Geological Voyage. - - - - - - - - 399
Raciborski's Elements of Diagnosis. - - - - 400

